# Successful Pregnancies, Births, and Children Development Following Oocyte Cryostorage in Female Cancer Patients During 25 Years of Fertility Preservation

**DOI:** 10.3390/cancers14061429

**Published:** 2022-03-10

**Authors:** Eleonora Porcu, Linda Cipriani, Maria Dirodi, Pierandrea De Iaco, Anna Myriam Perrone, Pier Luigi Zinzani, Mario Taffurelli, Claudio Zamagni, Patrizia Maria Ciotti, Leonardo Notarangelo, Nilla Calza, Giuseppe Damiano

**Affiliations:** 1 Infertility and IVF Unit, IRCCS Azienda Ospedaliero-Universitaria of Bologna, 40138 Bologna, Italy; linda.cipriani@aosp.bo.it (L.C.); maria.dirodi@aosp.bo.it (M.D.); patriziamaria.ciotti@aosp.bo.it (P.M.C.); leonardo.notarangelo@unibo.it (L.N.); nilla.calza@aosp.bo.it (N.C.); giuseppe.damiano@aosp.bo.it (G.D.); 2 Department of Medical and Surgical Sciences (DIMEC), University of Bologna, 40138 Bologna, Italy; 3 Division of Oncologic Gynecology, IRCCS Azienda Ospedaliero-Universitaria of Bologna, 40138 Bologna, Italy; pierandrea.deiaco@unibo.it (P.D.I.); myriam.perrone@unibo.it (A.M.P.); 4 Centro di Studio e Ricerca delle Neoplasie Ginecologiche (CSR), University of Bologna, 40138 Bologna, Italy; 5 Institute of Haematology “Seragnoli”, IRCCS Azienda Ospedaliero-Universitaria of Bologna, 40138 Bologna, Italy; pierluigi.zinzani@unibo.it; 6 Breast Unit, Department of Woman and Child, IRCCS Azienda Ospedaliero-Universitaria of Bologna, 40138 Bologna, Italy; mario.taffurelli@aosp.bo.it; 7 “Addari” Medical Oncology, IRCCS Azienda Ospedaliero-Universitaria of Bologna, 40126 Bologna, Italy; claudio.zamagni@aosp.bo.it

**Keywords:** oocyte cryopreservation, fertility preservation, child development, oncofertility

## Abstract

**Simple Summary:**

The study goal is to demonstrate that oocyte cryopreservation is a feasible and efficient option for fertility preservation in cancer patients through the comparison of in vitro fertilization treatments in nononcological patients.

**Abstract:**

The preservation of fertility in cancer patients is a crucial aspect of modern reproductive medicine. Amenorrhea and infertility often occur after cancer therapy, worsening the quality of life. Cryopreservation of oocytes in young cancer patients is a therapeutic option for preserving fertility. A prospective study was conducted on 508 cancer patients who underwent oocyte cryopreservation to preserve fertility between 1996 and 2021 including the COVID-19 pandemic period. Patients underwent ovarian stimulation, followed by egg retrieval, and oocytes were cryopreserved by slow freezing or vitrification. Sixty-four thawing/warming cycles were performed. Survival, fertilization, pregnancy, and birth rate over the thawing/warming cycles were obtained. The data were compared with those from a group of 1042 nononcological patients who cryopreserved supernumerary oocytes. An average of 8.8 ± 6.9 oocytes were retrieved per cycle, and 6.1 ± 4.2 oocytes were cryopreserved. With their own stored oocytes, 44 patients returned to attempt pregnancy. From a total of 194 thawed/warmed oocytes, 157 survived (80%). In total, 100 embryos were transferred in 57 transfer/cycles, and 18 pregnancies were achieved. The pregnancy rate per transfer and pregnancy rate per patient were 31% and 41%, respectively. No statistically significant differences were observed between oncological patients and nononcological patients. A total of 15 babies were born from oncological patients. Children born showed normal growth and development. One minor malformation was detected.

## 1. Introduction

The preservation of fertility in cancer patients has become mandatory in modern reproductive medicine and oncology. Amenorrhea and infertility often occur after cancer therapy, worsening the quality of life. It is well established that chemotherapy, especially alkylating agents, are gonadotoxic and may induce premature ovarian insufficiency (POI) [1,2]; therefore, the American Society of Clinical Oncology (ASCO) and the American Society for Reproductive Medicine (ASRM) recommend fertility preservation [3,4].

Oocyte cryopreservation is no longer experimental [5]; thus, egg storage is likely one of the best choices to preserve fertility in cancer patients [6,7,8,9,10,11]. Oocyte cryopreservation can be used alone or in association with GnRH analogue administration to improve patients’ opportunities [12]. Oocyte cryopreservation has a number of safety reports, and more than 3000 live births have been achieved with no evidence of an increase in child anomalies [13,14,15].

Regarding efficiency, cryopreserved oocytes’ fertilization, pregnancy, and live-birth outcomes are similar to those of fresh in vitro fertilization (IVF) [10,16].

In the 1990s, researchers at the University of Bologna began investigations into human oocyte cryopreservation [15,17,18,19,20,21,22,23,24,25,26,27,28], including its clinical application in cancer patients [1,6,7,9,29,30]; they oversaw the first successful birth from autologous frozen oocytes obtained before ovariectomy from a woman with a borderline ovarian tumor [31]. The present study reports the large number of cancer patients who underwent oocyte cryopreservation to preserve fertility and the follow-up of the 15 children born so far.

The study goal is to demonstrate that oocyte cryopreservation is a feasible and efficient option for fertility preservation in cancer patients through the comparison of in vitro fertilization treatments’ results in nononcological patients.

## 2. Materials and Methods

In total, 508 cancer patients underwent oocyte cryopreservation at the Infertility and IVF Unit, IRCCS Azienda Ospedaliero-Universitaria di Bologna, Italy, to preserve fertility before chemotherapy between 1996 and 2021 including the COVID-19 pandemic period. The average age of the patients diagnosed with cancer was 29.4 ± 4.0 (m ± sd), coinciding with the average age at the time of cryopreservation. Immediately after diagnosis and before chemotherapy, the patients were directed to the fertility preservation program. Of the patients, 108 had breast cancer, 83 had gynecological disease, 135 had hematological cancer, and 182 had other cancers (17 meningiomas, 12 ependymoma, 16 craniopharyngiomas, 21 thyroid tumors, 25 colorectal cancers, 7 lung cancers, 21 melanomas, 10 multiple myeloma, 6 Ewing sarcomas, 6 cancers of Wilms, 8 idiopathic myelofibroids, 9 essential thrombocythemia, 6 lymphoepithelioma, 6 sweat gland tumors, and 12 astrocytomas).

Patients were followed up for 1 to 25 years. At the starting visit and at the follow-up, menstrual rhythm was evaluated, and the ovarian reserve was determined by measuring serum AMH, FSH, LH, estradiol, and ultrasound antral follicle count (AFC). The occurrence of spontaneous pregnancy was assessed as well.

Oncological patients were compared with a group of 1042 nononcological infertile patients who underwent IVF procedures and had cryopreserved supernumerary oocytes in the same study period. The infertility causes were as follows: unexplained, 123 (11.8%); tubal occlusion, 83 (8.0%); anovulatory factor, 79 (7.6%); endometriosis, 118 (11.4%); male infertility, 265 (25.4%); and male and female infertility association, 374 (35.8%). Supernumerary oocytes were cryopreserved during the same study period.

In both oncological and nononcological patients, ovarian stimulation was performed with gonadotropins (urinary or follitropin α) associated with gonadotropin-releasing hormone (GnRH) agonist (leuprolide acetate) or antagonist (cetrorelix acetate). Ovulation was triggered with urinary or recombinant hCG.

Patients with hormone-sensitive tumors were stimulated with aromatase inhibitor cotreatment (letrozole 5 mg/die) to mitigate serum estradiol rise [29]. Monitoring of ovarian stimulation was performed with seriated estradiol blood tests and pelvic ultrasounds. When follicles reached a diameter of 16 mm and the estradiol serum levels were considered appropriate, ovulation was triggered with hCG 36 h prior to transvaginal ultrasound-guided oocyte retrieval.

Two cryopreservation techniques were utilized for oocyte preservation: slow freezing/rapid thawing and vitrification/warming. The first was utilized from 1996 to 2006, while the second one was utilized since 2006 up to today. Of all cycles, 69 were performed utilizing slow freezing and 439 were performed with vitrification. The slow-freezing protocol was previously reported in [17]; vitrification was performed according to Kuwayama’s protocol [32].

Briefly, slow freezing reduces intracellular ice formation and structural damage due to solute concentrations and osmotic stress, since oocytes are slowly cooled to below the freezing point using low concentrations of cryoprotectant agents (e.g., propanediol, sugars). During vitrification procedures, after a brief incubation into a solution containing a high concentration of cryoprotectant agents, oocytes are plunged directly into liquid nitrogen; in this way, they are “vitrified” without forming ice crystals. The term “vitrification” refers to the transformation from a liquid to a solid in the absence of crystallization, resulting in ‘‘glass formation” [33].

Forty-four patients returned to thaw their oocytes after recovery from cancer. They underwent endometrial preparation with 300 mcg/die hemihydrate estradiol patches from the first day of the menstrual cycle and continued until the pregnancy test was performed. Administration of micronized progesterone (1200 mg/die) vaginally was initiated 2 days prior to embryo transfer (day 2). Oocytes were thawed referring to the corresponding freezing protocol of slow freezing or vitrification and rapid thawing or warming, respectively.

If pregnancy was achieved, administration of estradiol and progesterone was maintained until gestational week 10.

In oocyte thawing cycles, all surviving metaphase II oocytes were inseminated through intracytoplasmic sperm injection, and embryos were cultured 2 days prior to intrauterine transfers.

One patient requiring transmyometrial embryo transfer due to squamous cervix cancer underwent a trachelectomy and lymphadenectomy, and embryo transfer was performed in general anesthesia using a Towako needle set (Cook).

Following successful pregnancies, deliveries and births were carried out by gynecologists and clinical psychologists, who assessed the course and outcome of the pregnancies through a structured questionnaire administered by phone calls [34].

The follow-up duration was in line with the data presented in the national and international registers [35]. In cases where an anomaly was suspected, direct consultations were conducted with family doctors and pediatricians.

Congenital anomalies and stages of psychophysical development were compared with the European guidelines for the surveillance of congenital anomalies (EUROCAT) and the new tables of child growth standards of the World Health Organization, respectively [35].

### Statistical Analysis

Data are expressed as mean ± sd (standard deviation).

Student’s *t*-test was used to compare age; FSH; AMH; AFC; follicle size; E2; oocytes retrieved, cryopreserved, and thawed/warmed; and embryos transferred.

The chi-square test was used for oocyte survival, fertilization, embryo transfer, pregnancy, miscarriage, and newborn rates.

*p*-Values of <0.05 were considered statistically significant.

All tests performed were validated through IBM SPSS Statistics (version 27.0).

Among all nononcological patients, comparing with oncological patients was performed by selecting those who cryopreserved supernumerary oocytes on the same day as an oncological patient.

## 3. Results

**Figure 1:**
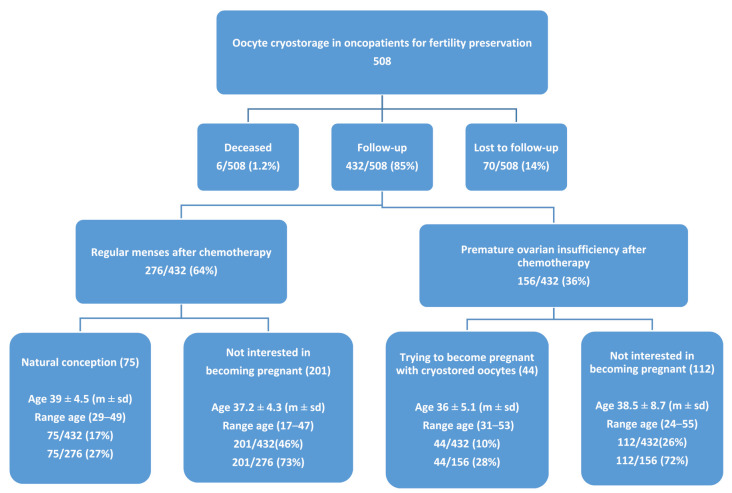
Flowchart of follow-up in oncological patients enrolled in the study.

Five hundred and eight patients were enrolled in the study, and a flowchart of the follow-up is presented in Figure 1.

Of the patients, 70/508 (14%) did not complete the follow-up required by the study, 6/508 (1.2%) deceased (2 had hematological cancer, 1 had astrocytoma, 1 had ependymoma, and 2 had breast cancer), and 432/508 (85%) completed the follow-up. In total, 276/432 (64%) patients resumed regular menstruation after chemotherapy, 75/432 (17%) conceived spontaneously, and 201/432 (46%) were not yet interested in becoming pregnant. Further, 156/432 (36%) had premature ovarian insufficiency (POI) after gonadotoxic treatment; among them, 44/156 (28%) returned to use their oocytes, while 112/156 (72%) were not yet interested in becoming pregnant.

The average time between oocyte cryopreservation and the last follow-up was 9.3 ± 6.8 years. In patients resuming menses, the cycle reappeared 10.5 ± 3.7 months after antineoplastic treatments ended.

Table 1 describes the characteristics of the oncological patients at the time of cryostorage (t_0_) and at follow-up (t_1_). Age was 29.4 ± 4 at cryostorage and 37.6 ± 5 at follow-up. At follow-up, a statistically significant reduction in ovarian reserve was observed, as assessed with AMH (0.7 ± 0.3 vs. 1.6 ±0.8), AFC (2.8 ± 1.6 vs. 7.1 ± 5), and FSH (34.5 ± 17.4 vs. 13.1 ± 8.1).

In Table 1, age and ovarian reserve parameters were also compared between patients who resumed menstruation after chemotherapy (Group a) and those who went into menopause (Group b). In Group a, a further division was made between patients who had a spontaneous conception (a1) and those who postponed the search for pregnancy (a2). In Group b, patients who thawed the oocytes (b1) were compared with those who went into menopause but did not require thawing (b2).

The reduction of ovarian reserve was by far lower in patients resuming normal menses after chemotherapy with respect to POI women.

Patients’ basal characteristic, results of hormonal stimulation, and oocytes’ features were compared with those of a group of nononcological infertile patients who cryopreserved supernumerary oocytes during the study period (Table 2).

No significant differences were seen between cancer and nononcological patients as regards age, ovarian reserve, number of follicles developed, peak estradiol levels, and number of oocytes retrieved and cryopreserved for similar periods of time.

No complications (OHSS, bleeding, infection) occurred after egg retrieval in either group.

Among the 156 women who had premature ovarian insufficiency (POI) after gonadotoxic treatment, 44 patients (28%) returned to use their oocytes and attempt a pregnancy after recovering from their disease and with the agreement of their oncologists. Among these patients, 18 had hematologic cancer, 11 had gynecological disease, 4 had breast cancer, and 11 had other cancers. The results of oocyte thawing/warming cycles were compared with those for the group of nononcological patients who used their supernumerary oocytes cryostored during the study period (Table 3). No significant differences were seen. Pregnancy and birth rates were almost the same in the two groups, as well have normal children growth and development. One single case of minor malformation was seen in the group of oncological patients. No malformations were detected in the nononcological group.

The numbers of oocytes cryostored, used, or still stored in oncological versus nononcological patients are reported in Table 4.

Table 5 shows the long-term follow-up of children conceived with cryopreserved oocytes in cancer patients. One single case of minor malformation (labiopalatoschisis) was registered. All the children had normal growth and development. Two children have already shown regular puberty.

Live birth case reports in cancer patients who preserved fertility through oocyte cryopreservation are presented in Table 6.

## 4. Discussion

In the field of assisted reproduction, fertility preservation has become a fundamental strategy, as it offers women the possibility of becoming pregnant after gonadotoxic treatments for cancer or age-related fertility decline. Oocyte cryopreservation has produced excellent results by allowing women who have undergone gonadotoxic treatments for cancer to become pregnant with embryos from their own oocytes. International guidelines prescribe that counseling should be offered to fertile women with a cancer diagnosis [46]. In some countries, including Italy, fertility preservation is free of charge for women facing cancer treatment involving the risk of sterility.

It is not so easy to establish a percentage of absolute infertility after antineoplastic treatments. One of the earliest studies was by Sauer et al. (1994) [47], who found that cancer survivors experience high spontaneous abortion rates following oocyte donation embryo transfers. Chow et al. (2016) [48] demonstrate that women who have received chemotherapy have lower pregnancy rates from natural conceptions and reduced live birth weights compared with sibling controls.

According to the results of the present study, the rate of premature ovarian insufficiency (POI) was 36%. On the other hand, in patients resuming regular cycles, only 27% had spontaneous natural pregnancy. Similar results were obtained in a previous study [1] with 29% of patients having menstrual irregularity after chemotherapy. The spontaneous pregnancy rate was 22% with healthy children in patients who resumed menstruation at the end of the treatment.

The low number of POI patients (44/156, 28%) requiring their stored oocytes to achieve a pregnancy is really surprising. The majority of POI women (112/156, 72%) did not attempt to become pregnant, and their oocytes are still stored in our cryobank.

This low rate of return may be related to the nature of cancer, its recovery time, and reproductive desire. Some patients do not survive until their healing. For those surviving, important personal evaluations, such as age and partner status, must be taken into account.

The low rate of patients returning to use their oocytes is consistent with that published in other studies [49,50].

Women’s age plays a crucial role in postchemotherapy infertility. In fact, age at the time of chemotherapy inversely correlates with the likelihood of postchemotherapy amenorrhea [1]. In addition, infertility is closely related to the ovarian reserve at the time of chemotherapy and the type of chemotherapy used.

Chemotherapeutic treatments can be gonadotoxic to primordial follicles, as they cause DNA strand breaks, induce apoptosis, and reduce stromal function within the ovary. The most damaging chemotherapies include alkylating chemotherapies (cyclophosphamide, busulfan, melphalan, procarbazine) and their combinations. In particular, the association of cyclophosphamide, methotrexate, and (F) 5-fluorouracil causes amenorrhea in 4% to 40% of women aged <35 years and 80% to 100% of women aged >35 years [51].

Radiation therapy may also have an impact on the fertility of cancer survivors depending on the cumulative dose of radiation, location of the treatment, and age of the patient. This reduction in ovarian reserve may cause immediate infertility and menopause after cancer treatment. Prepubescent girls have a better chance of achieving a healthy reproductive future after cancer treatment when compared with older women. Complete ovarian failure occurs with a dose of 20 Gy in women under 40 years of age, while the mean lethal dose for the human oocyte is estimated to be 2 Gy. In addition, pelvic radiation can permanently damage uterine elasticity and the musculature and vasculature of the endometrium, which may result in increased risk of miscarriage, midtrimester pregnancy loss, preterm birth, and low birth weight regardless of the age of exposure. One of the options to prevent POI due to pelvic radiation therapy (PRT) is ovarian transposition (OT), a simple technique in which the ovaries are placed outside the radiation field, thereby reducing the exposure to radiation and total dose of irradiation [2,36].

Centers offering a fertility preservation program should ensure close cooperation between reproductive gynecologists and oncologists to take care of the oncology patient as quickly as possible.

The present study reports a wide clinical experience and long-term follow-up of children after oocyte cryopreservation in cancer patients at risk of premature ovarian failure, supporting its use as an efficient fertility preservation technique.

The first live birth resulting from oocyte cryopreservation was reported by Chen in 1986 [37]. Since then, oocyte cryopreservation technology has significantly improved, making it a viable option for fertility preservation—particularly in oncology.

A total of 22 live births have been reported so far in cancer patients who preserved fertility through oocyte cryopreservation [7,10,11,31,38,39,40,41,42,43,44,45,50,52,53].

The present study adds 15 children born from cancer patients thanks to their oocytes cryostored before antineoplastic therapy. In addition, follow-up results are reassuring about children’s health.

In the present study, offspring’s main development endpoints—such as the teething, walking, language, and educational stages—were reported. The oldest babies are two female twins who are now nearly 14. The only malformation observed was one case of labiopalatoschisis in a female newborn, which was surgically corrected. All her perinatal scores were normal at birth and followed by normal growth.

In this study, assisted reproduction with cryopreserved oocytes appears to have the same efficient and safe results both in cancer and in nononcological patients.

In cancer patients, ovarian stimulation should be tailored according to age, basal AMH (anti-Müllerian hormone), ultrasound AFC (antral follicle count), and possible hormone-sensitive tumor malignancy type [54].

Aromatase inhibitor cotreatment during ovarian stimulation has been advocated to potentially mitigate the effects of elevated serum estradiol levels [29].

Available data show a substantially unchanged recurrence risk in patients who underwent ovarian stimulation with gonadotropins and an aromatase inhibitor. A prospective controlled study evaluated the recurrence risk in 79 women with breast cancer who underwent ovarian stimulation with gonadotropins and letrozole for embryo or oocyte cryopreservation compared with 136 breast cancer patients who underwent no fertility preservation procedure. The mean follow-up after chemotherapy was 23.4 months in the COS group and 33.05 months in the nononcological group. The recurrence rate and relapse-free survival were not significantly different between the two groups (respectively, *p* = 0.26 and *p* = 0.36; hazard ratio = 0.56) [55].

A retrospective cohort study by Turan [56] that involved 78 women aged ≤45 years diagnosed with stage ≤3 breast cancer evaluated the recurrence risk according to the number of ovarian stimulation cycles performed (1 vs. 2). No significant differences in recurrence rates were detected between the groups (0 of 17 in the two cycles vs. 2 of 49 in the single cycles) after a mean follow-up interval of 58.5 ± 13.6 months, whereas the mean numbers of oocytes retrieved and embryos generated were statistically significantly higher in the two-cycle group.

Hence, the ASCO guidelines now support the use of aromatase in women with hormone-sensitive cancers [3].

In accordance with the literature, our studies show that the use of an aromatase inhibitor during ovarian stimulation upholds oocyte cryopreservation as a viable technique for patients with hormone-sensitive tumors as well [29,30].

Timing of fertility preservation treatment in cancer patients is also particularly important as it should be performed before gonadotoxic treatments and after surgery.

Ovarian stimulation can be accomplished in 2–3 weeks, but standard ovulation protocols start within day 3 of the menstrual cycle. Recent data indicate that random stimulation can also be successful when the time to chemotherapy is reduced [3].

This study strengthens the existing literature on oncofertility and supports the importance of oocyte cryopreservation in oncological patients following the correct protocol and timing. Moreover, it provides new information about the long-term growth and development of babies born after oocyte cryopreservation in cancer patients, offering additional and crucial elements to be considered in fertility preservation counselling.

Proposals for future research include a longer follow-up of cancer patients’ offspring’s growth and long-term health.

To our knowledge, this is the first paper analyzing the long-term development of babies born after oocyte cryopreservation in mothers with cancer prior.

While being one of the longest-running studies on the topic, the results might be compromised by the evolution of technologies and protocols over the last 25 years.

Cryopreservation of oocytes has no longer been considered experimental since 2013 [5]. The vitrification technique has led to excellent results in terms of survival rates (80–95%) as well as high fertilization and implantation rates [41,57]. Cryopreservation of oocytes through vitrification is an effective, simple, safe, and efficient technique for fertility preservation in women who are about to start oncological treatments with gonadotoxic impact. Compared with slow freezing, oocyte vitrification provides better results, according to a recent meta-analysis [58].

A limitation to the study might be the small number of pregnancies and births, which, nevertheless, are due to the surprisingly low number of patients (44, i.e., 9%) choosing to use their oocytes stored before oncotherapies.

On the other hand, the numbers of pregnancies and births are not so small in themselves as these are the largest series of this kind ever obtained in a single study setting of cancer patients.

However, further confirmation of the present data in large cohorts is mandatory [59,60].

A limitation to the field could be the lack of a registry for oocyte cryopreservation. It would be useful to collect more data and fill registries with obstetrical outcomes of cancer survivors who used their previously cryopreserved oocytes.

## 5. Conclusions

Wide safety data regarding oocyte cryopreservation are currently being accumulated to improve counseling of patients desiring pregnancy. Fertility preservation using oocyte cryopreservation appears to be a viable option for cancer patients at risk of ovarian failure, allowing women to achieve pregnancies and births and improve their quality of life [35].

Collaboration among reproductive physicians, biologists, endocrinologists, oncologists, surgeons, psychologists, and gynecologists is necessary to ensure the best fertility preservation program for each patient.

## Figures and Tables

**Table 1:** Follow-up of oncological patients: comparison of age and ovarian reserve between the time of cryostorage and follow-up.

		Cryostorage (t_0_)					Follow-Up (t_1_)				*p* t_0_ vs. t_1_
		No. of Patients (%)	AGE	AFC	AMH	FSH	AGE	AFC	AMH	FSH	
**Total Patients**		508	29.4 ± 4.0	7.1 ± 5.0	1.6 ± 0.8	13.1 ± 8.1	37.6 ± 5.6	2.8 ± 1.6	0.7 ± 0.3	34.5 ± 17.4	Age t_0_–t_1_ 0.001 AFC t_0_–t_1_ 0.001 AMH t_0_–t_1_ 0.001 FSH t_0_–t_1_ 0.001
**Regular menses after chemotherapy 276/432 (64%) (a)**		276	28.5 ± 5.2	8.7 ± 2.2	1.6 ± 0.6	7.8 ± 4.5	38.1 ± 4.2	4.3 ± 2.8	1.3 ± 0.3	6.8 ± 2.9	at t_0_ Age a vs. b 0.001 AFC a vs. b 0.001 AMH a vs. b 0.001 FSH a vs. b 0.001
	**Natural conception (a1)**	75/276 (27%)	29 ± 4.8	9.3 ± 3.8	1.9 ± 0.6	7.4 ± 3.2	39 ± 4.5	5.3 ± 3.2	1.5 ± 0.4	6.7 ± 4.5	Age t_0_–t_1_ 0.001 AFC t_0_–t_1_ 0.001 AMH t_0_–t_1_ 0.001 FSH t_0_–t_1_ 0.274
	**Not interested in becoming pregnant (a2)**	201/276 (73%)	28.6 ± 6.7	8.2 ± 4.7	1.5 ± 0.9	9.5 ± 6.6	37.2 ± 4.3	4.5 ± 2.1	1.2 ± 0.5	7.2 ± 3.3	Age t_0_–t_1_ 0.001 AFC t_0_–t_1_ 0.001 AMH t_0_–t_1_ 0.001 FSH t_0_–t_1_ 0.001
**POI after chemotherapy 156/432 (36%) (b)**		156	31.5 ± 4.8	7.2 ± 1.7	1.1 ± 0.7	9.1 ± 3.2	37.2 ± 4.9	0.4 ± 0.3	0.2 ± 0.1	63.2 ± 17.0	at **t_1_** Age a vs. b 0.044 AFC a vs. b 0.001 AMH a vs. b 0.001 FSH a vs. b 0.001
	**Not interested in becoming pregnant (b1)**	112/156 (72%)	32 ± 6.7	7.2 ± 1.8	1.3 ± 0.9	9.02 ± 5.4	38.5 ± 8.7	0.7 ± 0.5	0.03 ± 0.01	72.0 ± 43.0	Age t_0_–t_1_ 0.001 AFC t_0_–t_1_ 0.001 AMH t_0_–t_1_ 0.001 FSH t_0_–t_1_ 0.001
	**Trying to become pregnant with cryostored oocytes (b2)**	44/156 (28%)	29.4 ± 4.0	7.3 ± 2.1	1.4 ± 0.8	9.3 ± 2.8	36.0 ± 5.1	0.5 ± 0.3	0.23 ± 0.11	56.0 ± 23.0	Age t_0_–t_1_ 0.001 AFC t_0_–t_1_ 0.001 AMH t_0_–t_1_ 0.001 FSH t_0_–t_1_ 0.001
**Lost to follow-up (70)**		70/508 (14%)	29.7 ± 3.5	8.6 ± 5.1	1.8 ± 0.7	7.7 ± 3.2	-	-	-	-	
**Deceased (6)**		6/508 (1.2%)	29.3 ± 2.1	5.1 ± 1.8	0.9 ± 0.3	10.1 ± 2.3	-	-	-	-	

POI—premature ovarian insufficiency; AFC—antral follicle count; AMH—anti-Müllerian hormone.

**Table 2:** Patients’ basal characteristic and oocyte features in oncological patients vs. nononcological patients.

	Oncological Patients	Nononcological Patients	*p*
Patients (*n*)	508	1042	
Age (years) (m ± sd)	29.4 ± 4.0	30.0 ± 6.8	0.066
FSH (IU/L) (m ± sd)	13.1 ± 8.1	12.4 ± 7.7	0.099
AMH (ng/mL) (m ± sd)	1.6± 0.8	1.6 ± 0.9	1.000
AFC *n* (m ± sd)	7.1 ± 5.0	7.3 ± 2.0	0.263
Length of storage (years) (range) (m ± sd)	1–25 (5.6 ± 3.2)	1–25 (4.8 ± 3.7)	0.210
FSH administrated (IU) (m ± sd)	2630 ± 1402	2750 ± 1305	0.098
Follicles > 16 mm *n* (m ± sd)	8.0 ± 3.0	7.7 ± 3.4	0.091
E2 max (pg/mL) (m ± sd)	1280 ± 645	1345 ± 1070	0.207
Oocytes retrieved *n* (m ± sd)	3604 (8.8 ± 6.9)	7644 (8.5 ± 6.8)	0.067
Oocytes cryopreserved *n* (m ± sd)	2966 (6.1 ± 4.2)	5046 (5.9 ± 4.8)	0.060

**Table 3:** Results of oocyte thawing/warming cycles in oncological patients vs. nononcological patients.

	Oncological Patients	Nononcological Patients	*p*
Patients (*n*)	44	870	
Age at cryopreservation years (m ± sd)	29.4 ± 4.0	30.0 ± 6.8	0.562
Age at oocyte thawing/warming years (m ± sd)	36.0 ± 5.1	37.1 ± 4.2	0.094
Thawing/warming cycles (*n*)	64	1315	
Length of storage years (range) (m ± sd)	2–15 (5.0 ± 3.8)	2–15 (4.8 ± 3.7)	0.146
Thawed/warmed oocytes *n* (m ± sd)	194 (3.7 ±1.9)	4208 (3.5 ± 1.8)	0.131
Oocytes survived (%)	157 (80.9)	3172 (75.4)	0.094
Oocytes fertilized (%)	101/138 (73.2)	2172/2793 (77.8)	0.249
Embryo transfers (%)	57/64 (89.1)	1165/1294 (90.0)	0.969
Embryo transferred *n* (m ± sd)	100 (1.7 ± 0.7)	2044 (1.8 ± 0.6)	0.107
Pregnancies *n*	18	361	0.958
Births *n*	13	283	
Newborns *n*	15	302	0.772
Miscarriages *n* (%)	4 (22)	78/361 (21.6)	0.817
Pregnancy per patient (%)	18/44 (41.0)	361/870 (41.4)	0.936
Pregnancy per cycle (%)	18/64 (28.1)	361/1315 (27.4)	0.980
Pregnancy per transfer (%)	18/57 (31.5)	361/1165 (31.0)	0.958
Births per patient (%)	13/44 (29.9)	283/870 (32.5)	0.805
Births per cycle (%)	13/64 (20.3)	283/1315 (21.5)	0.941
Births per transfer (%)	13/57 (22.8)	283/1165 (24.2)	0.866
Newborns per patient (%)	15/44 (34.1)	302/870 (34.7)	0.938
Newborns per cycle (%)	15/64 (23.4)	302/1315 (23.9)	0.949
Newborns per transfer (%)	15/57 (26.3)	302/1165 (26.0)	0.999

**Table 4:** Oocytes cryopreserved, thawed, or still stored in oncological vs. nononcological patients.

	Oncological Patients	Nononcological Patients	*p*
Oocytes cryopreserved *n* (m ± sd)	2966 (6.1 ± 4.2)	5046 (5.9 ± 4.8)	0.060
Thawed/warmed oocytes *n* (m ± sd)	194 (3.7 ± 1.9)	4208 (3.5 ± 1.8)	0.131
Oocyte still in storage *n* (m ± sd)	2772 (2.5 ± 1.1)	838 (2.4 ± 1.4)	0.031

**Table 5:** Long-term follow-up of children conceived with oocytes cryopreserved in cancer patients.

Sex	Current Age (y/mths)	Mother’s Cancer Type	Slow-Freezing/Vitrification	Length of Storage (y)	Length of Gestation (Weeks)	Delivery	Twins	Apgar Score 1 min	Apgar Score 5 min	Weight (g)	Length (cm)	Head Circumference (cm)	Malformations	Current Height (cm)	Current Weight (kg)	Teething (mths)	Walking (mths)	Language (mths)	Puberty (y)	Educational Stage
M	7 y 6 mths	Breast cancer	Slow	3	39	CS		10	10	3320	52	32	-	134	29	8	12	24	-	1° grade
F	7 y 4 mths	Breast cancer	Vitri	2	37	CS	Yes	9	10	2050	47	32	-	132	25	9	10	20	-	1° grade
F	7 y 4 mths	Breast cancer	Vitri	2	37	CS	Yes	9	10	2045	48	33	-	130	26	9	11	18	-	1° grade
F	10 mths	Breast cancer	Vitri	3	39	CS		8	9	3670	48	34	-	70	12					
F	6 y 5 mths	BOT	Vitri	3	38	CS		9	10	3900	50	34	-	115	21	10	10	13	-	1° grade
M	4 y 1 mths	BOT	Vitri	2	39	CS		10	10	3250	48	33	-	99	15	6	14	20	-	Kindergarten
F	10 y	BOT	Slow	5	38	CS		9	10	2980	48	32	Labiopalatoschisis	140	32	8	12	18	-	1° grade
F	13 y 5 mths	BOT	Slow	4	34	CS	Yes	9	10	2120	48	33	-	152	48	8	14	16	11	Middle school
F	13 y 5 mths	BOT	Slow	4	34	CS	Yes	9	10	2090	48	32	-	148	50	8	13	16	12	Middle school
M	7 y	Endometrial cancer	Slow	4	40	CS		10	10	3850	50	34	-	133	30	12	9	14	-	1° grade
M	1 y 10 mths	NHL	Slow	6	39	CS		9	10	3130	49	33	-	104	17	12	12	Few words	-	Kindergarten
M	2 y	HL	Vitri	7	38	SD		9	10	2575	48	32	-	85	16	10	12	12	-	-
M	2 y 1 mths	HL	Vitri	5	39	SD		10	10	3125	49	32	-	87	14	11	11	14	-	-
F	2 y 4 mths	HL	Vitri	4	40	CS		10	10	3320	55	34	-	88	13	9	14	14	-	-
M	1 mths	BOT	Vitri	3	39	SD		9	10	3150	50	33	-	87	6					

CS—caesarian section; SD—spontaneous delivery; BOT—borderline ovarian tumor; HL—Hodgkin lymphoma; NHL—non-Hodgkin lymphoma; y—years; mths—months.

**Table 6:** Live birth case reports of the literature in cancer patients who preserved fertility through oocyte cryopreservation.

Authors	Malignancy	Age at Cryopreservation	Cryopreservation Technique	Age at Thawing/Warming	N MII Oocytes Cryopreserved	No. of Embryos Transferred	Pregnancies	Delivery	No. of Live Births	Sex
Yang et al., 2007 [36]	Hodgkin lymphoma	27	Slow freezing	33	13	3 + 3 + 3 (gestational carrier)	Single	-	1	M
Porcu et al., 2008 [31]	Borderline ovarian tumor	27	Slow freezing	31	7	3	Twins	CS	2	F, F
Sanchez-Serrano et al., 2010 [37]	Breast cancer	36	Vitrification of oocytes after stimulation of ovarian tissue transplanted	36	9	2	Twins	CS	2	M, M
Kim et al., 2011 [38]	Chronic myeloid leukemia	22	Vitrification	31	7	2	Single	CS	1	M
Garcia Velasco et al., 2013 [39]	Hodgkin lymphoma	33	Vitrification	35	4	2	Single	VD	1	M
Martinez et al., 2014 [40]	Breast cancer	30	Vitrification	33	5	2	Single	CS	1	M
Martinez et al., 2014 [40]	Breast cancer	33	Vitrification	38	3	2	Single	VD	1	F
Martinez et al., 2014 [40]	Breast cancer	37	Vitrification	40	8	2	Single	CS	1	M
Martinez et al., 2014 [40]	Hodgkin lymphoma	33	Vitrification	35	4	2	Single	VD	1	F
Alvez Da Motta et al., 2014 [41]	Breast cancer	36	Vitrification	41	28	3 + 3	Single	CS	1	-
Alvarez et al., 2014 [42]	Invasive ovarian cancer	28	Vitrification	29	14	2	Heterotopic	CS	1	M
Druckenmiller et al., 2016 [11]	Gynecological cancer	28	Vitrification	-	8	2 (gestational carrier)	Twins	-	2	-
Druckenmiller et al., 2016 [11]	Breast cancer	33	Slow freezing	-	8	2 (gestational carrier)	Single	-	1	-
Druckenmiller et al., 2016 [11]	Breast cancer	39	Slow freezing	-	8	2	Single	-	1	-
Druckenmiller et al., 2016 [11]	Breast cancer	40	Slow freezing	-	8	2	Single	-	1	-
Perrin et al., 2016 [43]	Hodgkin lymphoma	29	Vitrification	31	5	2	Single	VD	1	F
Doyle et al., 2016 [44]		-	Vitrification	-	-	-	Single	-	1	-
Specchia et al., 2019 [45]	Breast cancer	35	Vitrification	40	9	3 + 1	Single	-	1	-
Specchia et al., 2019 [45]	Breast cancer	36	Vitrification	40	13	2	Single	-	1	-

## Data Availability

The datasets generated and/or analyzed during the current study are available from the corresponding author on reasonable request.
